# Comparison of incubation period distribution of human infections with MERS-CoV in South Korea and Saudi Arabia

**DOI:** 10.1038/srep35839

**Published:** 2016-10-24

**Authors:** Victor Virlogeux, Vicky J. Fang, Minah Park, Joseph T. Wu, Benjamin J. Cowling

**Affiliations:** 1 Department of Biology, Ecole Normale Supérieure de Lyon, Lyon, France; 2 WHO Collaborating Centre for Infectious Disease Epidemiology and Control, School of Public Health, Li Ka Shing Faculty of Medicine, The University of Hong Kong, Hong Kong Special Administrative Region, China; 3 Cancer Research Center of Lyon, UMR Inserm U1052, CNRS 5286, Lyon, France

## Abstract

The incubation period is an important epidemiologic distribution, it is often incorporated in case definitions, used to determine appropriate quarantine periods, and is an input to mathematical modeling studies. Middle East Respiratory Syndrome coronavirus (MERS) is an emerging infectious disease in the Arabian Peninsula. There was a large outbreak of MERS in South Korea in 2015. We examined the incubation period distribution of MERS coronavirus infection for cases in South Korea and in Saudi Arabia. Using parametric and nonparametric methods, we estimated a mean incubation period of 6.9 days (95% credibility interval: 6.3–7.5) for cases in South Korea and 5.0 days (95% credibility interval: 4.0–6.6) among cases in Saudi Arabia. In a log-linear regression model, the mean incubation period was 1.42 times longer (95% credibility interval: 1.18–1.71) among cases in South Korea compared to Saudi Arabia. The variation that we identified in the incubation period distribution between locations could be associated with differences in ascertainment or reporting of exposure dates and illness onset dates, differences in the source or mode of infection, or environmental differences.

South Korea experienced the largest outbreak of Middle East respiratory syndrome coronavirus (MERS-CoV) infections outside the Arabian Peninsula with 186 laboratory-confirmed cases from May to July 2015^1^. As of 26 April 2016, a total of 1,728 laboratory-confirmed cases of MERS-CoV infection have been diagnosed globally and reported to the World Health Organization since the first reported case in Saudi Arabia in 2012, of which more than 1,000 cases have occurred in Saudi Arabia^1^,^2^,^3^,^4^. Estimation of the incubation period distribution, which is defined as the period between exposure/infection and the appearance of the first symptoms^5^, is a key parameter in the transmission dynamics, and forms part of the case definition^6^, is used to define the appropriate quarantine period, and is one of the parameters used in mathematical modeling studies to predict the impact of different control strategies^7^. There are a few published reports of the incubation period distribution of MERS-CoV infection, with a median incubation period varying from 5.2 days (95% confidence interval, 1.9–14.7 days) in the Middle East^8^ to 6.0 days (95% confidence interval, 4–7 days) and 6.3 days (95% credibility interval, 5.7–6.8 days) in the recent outbreak in South Korea^9^,^10^. The objective of our study was to describe alternative approaches for estimation of the incubation period of MERS-CoV infection and to investigate whether there was variability in the incubation period by age, sex and geographic location.

## Results

Data on defined exposure periods and onset dates for cases in South Korea of the 2015 outbreak were available from 115 (63%) of all 186 patients diagnosed with laboratory-confirmed MERS-CoV infection, and we identified published data on exposure periods and onset dates for 34 patients of the 1,456 patients in Saudi Arabia as of 4 May 2016^2^,^11^,^12^,^13^,^14^. The characteristics of patients from Korea and from Saudi Arabia are reported in Table 1. Among patients with exposure data, the mean age was 54 years and 63% were males, and the age and sex of patients were similar in South Korea and in Saudi Arabia. The risk of death was much higher in the cases from Saudi Arabia (16/34; 47%) compared to the cases from South Korea (26/115; 23%) (p = 0.01, chi-squared test).

**Figure 1:**
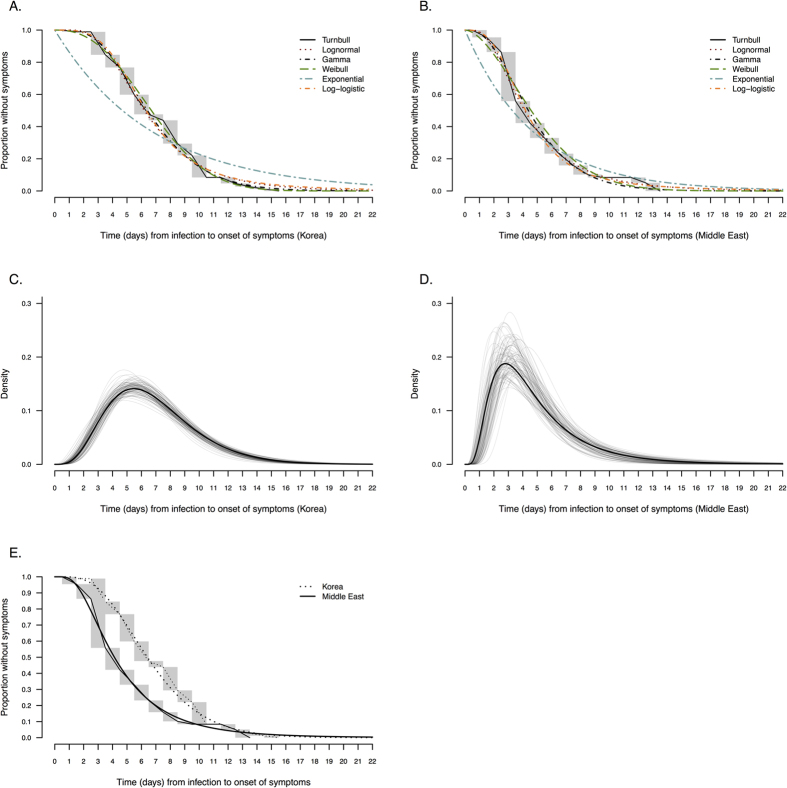
Comparison of nonparametric and parametric estimates of the incubation period distribution in cases of MERS-CoV infection in South Korea and Saudi Arabia. Panel (A,B) compare the Turnbull nonparametric estimate of the incubation period distribution with the fitted lognormal, Weibull, gamma, loglogistic and exponential distributions using data from (**A**) South Korea (n = 115) and Saudi Arabia (n = 34). Panel (C,D) present the probability density function of the parametric model with the best BIC value for the cases in South Korea (gamma distribution) and in Saudi Arabia (lognormal distribution). The solid line represents the uncertainty range estimated by bootstrapping with 1,000 resamples. Panel (E) compares the nonparametric (Turnbull) and parametric estimates of the incubation period distribution in South Korea (gamma distribution) and in Saudi Arabia (lognormal distribution).

Figure 1A,B compare alternative parametric models with the non-parametric maximum likelihood estimator. Visual inspection of the parametric curves against the Turnbull estimate in Fig. 1A,B confirm that all of the two-parameter distributions provided reasonable fits, while the one-parameter exponential distribution was inferior. Among the cases in South Korea, the gamma and Weibull parametric models (Fig. 1C) had the best BIC value with an estimated mean of 6.9 days (95% credibility interval: 6.3–7.5) (Table 2). Among cases from Saudi Arabia, the lognormal distribution (Fig. 1D) had the best BIC value with an estimated mean of 5.0 days (95% credibility interval: 4.0–6.6) (Table 2). The other fitted two-parameter distributions had generally similar means, with 95^th^ percentiles in the range 10–14 days and 99^th^ percentiles in the range 14–22 days (Table 2). Except for the exponential distribution, the various two-parameter distributions had similar BIC values among the cases in each location.

Since a lognormal distribution gave a good fit to the data in both locations, we pooled information from both locations and fitted a log-linear regression model to the data. Using that model, we found that the mean incubation period was 1.40 times longer (95% credibility interval: 1.15–1.71) among cases in South Korea compared to Saudi Arabia without adjustment, and the estimate was almost the same after adjustment for age and sex (Table 3).

## Discussion

Using all available data for the recent outbreak of MERS-CoV infections in South Korea, and published data from cases in Saudi Arabia, we estimated that the mean incubation period was 6.9 days for cases in South Korea and 5.0 days for cases in Saudi Arabia. In various parametric models, the 95^th^ percentiles were in the range 10–14 days, which is consistent with the currently used case definitions^6^. While it is difficult to estimate the right hand tail of the incubation period distribution based on small sample sizes, we estimated the 99^th^ percentile could be as long as 14–22 days and this indicates that long incubation periods are possible. In South Korea, one of the 186 cases was reported to have an incubation period of 21 days or longer, although it has been suggested that immunosuppression in that person could potentially have delayed the onset of symptoms^15^.

Our estimates for the incubation period distribution of MERS-CoV infections in Saudi Arabia are consistent with the previous estimates of Assiri *et al*.^8^. in a hospital outbreak in the eastern province of Saudi Arabia based on 23 cases with an estimated median incubation period of 5.2 days (95% confidence interval: 1.9–14.7 days). Our estimates for cases in South Korea are also close to other reports with an estimated mean incubation period of 6.7 days (95% credibility interval: 6.1–7.3 days)^9^, and a median of 6 days in one hospital^9^,^10^.

We found a significant difference in mean incubation periods between the cases in South Korea and in Saudi Arabia (Table 3). This difference could be related to the transmission dynamics of MERS-CoV infection with only secondary cases and longer transmission chains in the outbreak in South Korea^9^, compared with cases in Saudi Arabia included in this study where a majority (74%) came from the same hospital^8^ where it has already been shown that the transmission chain was shorter with multiple separate animal-to-human infections^16^,^17^. Potential direct transmission could be related to a higher infecting dose and higher virulence of the strain that could lead to a shorter incubation period^18^. A recent studies on MERS-CoV transmission during the outbreak in South Korea reported different estimates of the incubation period depending on the intensity of exposure and/or inoculation route^19^. Indeed, the authors showed that the incubation period was significantly shorter among patients that were exposed to the index case in the same zone of the emergency room (median: 5 days; interquartile range (IQR): 4–8 days) compared with patients from different zones (median: 11 days; IQR: 6–12 days). These results strengthen the hypothesis that a higher infecting dose could have been transmitted by the index case leading to a shorter incubation period compared with cases associated with “indirect” transmission that may have been responsible for transmission in different zones of the emergency room. Further investigation on human-to-human and human-to-animal transmission dynamics would improve our understanding of the potential role of the exposure route on the incubation period. It is also possible that this difference is an artifact of different approaches to data collection or reporting in South Korea and in Saudi Arabia.

Our study had some limitations. First, we did not have access to original patient records, and our data on MERS-CoV infections in South Korea were based on publicly available information while we relied on published data for a relatively small number of cases in Saudi Arabia. There is a potential concern that symptoms and symptom onset dates might be reported differentially in the two locations. In the cases of MERS-CoV infection reported by Assiri *et al*. from Saudi Arabia, 20/23 cases had fever on the day of symptom onset^11^. In the outbreak in South Korea, fever was part of the case definition and symptom onset may reflect the date of onset of fever rather than other symptoms^20^,^21^. Secondly, regarding patients from Saudi Arabia, only 34 patients among the 1,456 patients (2%) diagnosed with MERS-CoV infection since 2012 in Saudi Arabia had publicly-available exposure data and most of these patients (25/34, 73%) came from the same hospital^8^. However, a larger proportion of patients from the outbreak in South Korea (N = 115; 63%) had publicly-available exposure data. Third, the outbreak in South Korea occurred 2 years after the first officially announced case of MERS-CoV infection in Saudi Arabia during which time the virus may have evolved somewhat, including changes in the transmission and pathogenesis characteristics. Fourth, no information about inoculation route was available for the patients included in this study as the data were retrieved from publicly available data or published studies.

In conclusion, accurate and rapid estimates of the length of incubation period are required during an outbreak to advise public health policy, to specify case definitions, and to facilitate robust mathematical modeling. In this paper, we assessed precisely the length of incubation period of MERS-CoV infections using two different datasets from Saudi Arabia and from South Korea and showed that the incubation period of MERS-CoV infections appeared to vary depending on the location of the outbreak.

## Methods

### Sources of data

For the outbreak in South Korea, we retrieved publicly available data from multiple sources, including the Korea Center for Disease Control and Prevention, the Korean Ministry of Health and Welfare, the World Health Organization, and local Korean news reports to compile a line list of all confirmed cases that had been reported by 27 July 2015. We used the most updated information from official reports that have been published by the Center for Disease Control and Prevention and the Ministry of Health and Welfare on a daily basis during the outbreak. The official reports included a brief description of each of all confirmed cases, including demographic characteristics (e.g., age and sex), dates of exposure, onset of symptoms and outcome. The information on exposure was mostly recorded as intervals of 2 to 15 days during which transmission was thought to have occurred rather than exact dates of presumed transmission.

Information on cases of MERS-CoV infection in the Middle East were retrieved from four published studies that provided individual patient data from Saudi Arabia^11^,^12^,^13^,^14^. We selected only the cases with available exposure information and collected data including demographic characteristics (e.g. age and sex), dates of exposure and onset of symptoms, geographical location of the exposure, and final outcome. For both locations, the day of symptoms onset was defined as the day when clinical symptoms related to MERS-CoV infection first occurred, including non-specific symptoms such as fever, chills, shortness of breath, cough, sputum, sore throat, myalgia, diarrhea, nausea and vomiting^1^,^11^,^12^,^13^,^14^,^20^,^21^.

### Statistical analyses

The incubation period T_k_ for each case *k* is defined as T_k_ = S_k_–X_k_, where S_k_ is the symptom onset time and X_k_ the infection time. Infection events are rarely observed but rather interval-censored. If case *k* reported that exposure to infection occurred in a period between times L_k_ and U_k_, where L_k_≤X_k_≤U*k*, the incubation time therefore is bounded by the interval (S_k_-U_k_, S_k_-L_k_). These interval-censored data are a special type of survival data, and it is possible to “reverse” the time axis considering S_k_ as the origin and X_k_ as the outcome time, if the density function for infection is uniform in chronologic time^22^. This condition should be reasonable here in the setting of MERS-CoV infections, with each exposure interval being relatively short, and reversing the time axis allowed us to use standard approaches for interval-censored data. We added +0.5 to each upper bound and −0.5 to each lower bound to give appropriate intervals in continuous time and to account account for uncertainty in the reported exposure times^23^. For example, an exposure that was reported two and three days before illness onset would be written as an incubation period censored on the interval (1.5, 3.5) instead of (2,3).

To deal with interval-exposure data, the most basic approach is to impute the infection dates as the midpoint of exposure intervals, but this leads to overestimation of the incubation period distribution, which tends to be right-skewed^24^. Non-parametric estimation of a distribution based on interval-censored data can be done with the generalized non-parametric maximum likelihood estimator developed by Turnbull^25^. The incubation period can often be appropriately characterized by different parametric distributions that have been previously used such as gamma^9^,^26^, Weibull^9^,^27^,^28^, lognormal^9^,^18^, log-logistic, and exponential distributions. We fitted five different distributions and estimated the parameters of each distribution using Markov Chain Monte Carlo (MCMC) in a Bayesian framework. The incubation period distribution was estimated using first the interval-censored data and compared between the different parametric models (using Bayesian Information Criterion) and the Turnbull non-parametric estimate^25^.

To evaluate potential factors such as age, sex and geographic location that could be associated with the length of the incubation period, we used a linear regression model on the log of the incubation period (assuming that incubation periods generally followed lognormal distributions), which can also be referred to as a log-linear model. The multiple linear regression model used in this study is based on the following equation:


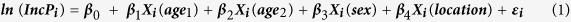


where *IncP*_i_ is the length of incubation period for individual *i, β*_*i*_’s are the regression coefficients, estimated with MCMC using flat priors, *X*_*i*_ ’s the explanatory variables labeled directly in the equation above and **ε**_**I**_the disturbance factor, normally distributed, independently and identically with E(*εi*) = 0 and *V*(*εi*) = *σ*^2^ for all *i*. We used two different approaches to estimate model parameters, including an exact likelihood method and a resampling method^29^.

### Approach 1: exact likelihood approach

The equation (1) above can be written as:


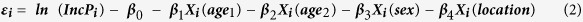


and consequently using equation (2) we can define the following pdf of the normal distribution:


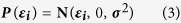


We defined the probability *q*_*i*_ as:


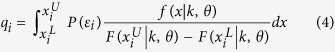


where 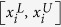 is the range of incubation period for case *i* and where (*k*, *θ*) is the couple of parameters of the gamma distribution.

We estimated *θ* = (*β*_*0*_, *β*_*1*_, *β*_*2*_, *β*_*3*_, *β*_*4*_, *k*, *θ, σ*^2^) simultaneously using MCMC and the following likelihood:


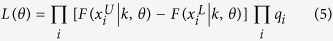


where *f* and F are the pdf and cdf of the gamma distribution with parameters k and θ, respectively.

### Approach 2: resampling approach

We defined another multiple linear regression model using incubation times resampled from the 10,000 posterior samples. In this approach, the probability *P(ε*_*i*_) was similarly defined as in equation (3) and for each patient with interval-censored exposure data, we estimated 10,000 posterior samples for the incubation time using MCMC in order to simulate the incubation period distribution for each patient. We used the same likelihood as defined in equation (5) using the resampled incubation time for each patient.

In this analysis and the analyses described above, we used a Metropolis-Hasting algorithm, specified flat priors for each parameter, and drew 10,000 samples from the posterior distributions after a burn-in of 5,000 iterations. All analyses presented here were conducted using R version 3.2.2 (R Foundation for Statistical Computing, Vienna, Austria).

## Additional Information

**How to cite this article**: Virlogeux, V. *et al*. Comparison of incubation period distribution of human infections with MERS-CoV in South Korea and Saudi Arabia. *Sci. Rep.* **6**, 35839; doi: 10.1038/srep35839 (2016).

## Figures and Tables

**Table 1:** Characteristics of cases of MERS-CoV infection in South Korea and Saudi Arabia.

Case characteristics	South Korea	Saudi Arabia	Overall
Sample size, n (%)	115 (77%)	34 (23%)	149
Age, n (%)			
0–45 years old	32 (28%)	10 (29%)	42 (28%)
46–59 years old	39 (34%)	15 (44%)	54 (36%)
>59 years old	44 (38%)	9 (26%)	53 (36%)
Male sex, n (%)	70 (61%)	24 (71%)	94 (63%)
Fatal cases, n (%)	26 (23%)	16 (47%)	42 (28%)

**Table 2:** Alternative parametric estimates of the mean of the incubation distribution of MERS-CoV infection based on all available data.

Distribution	Mean (days)		95th percentile (days)		99th percentile (days)		BIC	DIC
	Estimate	95% CrI^1^	Estimate	95% CI^1^	Estimate	95% CI^1^		
South Korea (n = 115)								
Gamma	6.9	(6.3–7.5)	12.7	(11.5–14.4)	16.1	(14.3–18.7)	418	412
Weibull	6.9	(6.3–7.5)	12.2	(11.2–13.5)	14.5	(13.2–16.4)	418	412
Lognormal	7.0	(6.3–7.7)	13.7	(11.9–16.1)	19.0	(16.0–23.3)	423	417
Log-logistic	7.2	(6.5–8.0)	14.2	(12.3–16.9)	22.3	(18.3–28.5)	425	419
Exponential	6.8	(5.7–8.2)	20.4	(17.1–24.5)	31.3	(26.3–37.6)	505	498
Saudi Arabia (n = 34)								
Lognormal	5.0	(4.0–6.6)	11.4	(8.5–17.5)	17.5	(12.0–30.6)	124	121
Log-logistic	5.1	(4.1–6.8)	11.2	(8.1–17.7)	19.6	(12.3–38.3)	124	121
Gamma	4.9	(4.0–6.0)	10.0	(8.0–13.3)	13.3	(10.3–18.6)	125	122
Weibull	5.0	(4.0–6.2)	10.7	(8.7–14.5)	13.8	(10.9–20.0)	127	124
Exponential	4.6	(3.4–6.6)	13.9	(10.3–19.7)	21.4	(15.9–30.3)	137	132

^1^95% credibility intervals (CrIs) calculated from the parameters posterior distributions using MCMC with 10,000 repetitions.

BIC: Bayesian Information Criterion. DIC: Deviance Information Criterion.

**Table 3:** Factors associated with the incubation period of MERS-CoV infection.

Factors	Coefficient exp(β) (95% CrI)^1^	Coefficient exp(β) (95% CrI)^1^
Approach 1: continuous incubation period using exact likelihood		
Age		
0–45 years old	—	1.00
46–59 years old	—	1.19 (0.99–1.43)
>59 years old	—	1.13 (0.94–1.40)
Sex		
Female	—	1.00
Male	—	0.89 (0.76–1.04)
Location		
Saudi Arabia	1.00	1.00
South Korea	1.42 (1.18–1.71)	1.40 (1.18–1.68)
Approach 2: continuous incubation period^2^		
Age		
0–45 years old	—	1.00
46–59 years old	—	1.20 (0.98–1.47)
>59 years old	—	1.13 (0.93–1.40)
Sex		
Female	—	1.00
Male	—	0.89 (0.76–1.04)
Location		
Saudi Arabia	1.00	1.00
South Korea	1.40 (1.15–1.71)	1.41 (1.17–1.72)

^1^The coefficients (β) of the multiple linear regression were estimated using Markov Chain Monte Carlo (10,000 runs) with incubation period as the outcome variable and age, sex and location as predictors. Moreover, 10,000 samples from the posterior distributions of the incubation periods T for each patient estimated with were used here in the multiple regression model.

^2^10,000 samples of the incubation periods T for each patient were drawn using MCMC.
